# The ClpP protease homologue is required for the transmission traits and cell division of the pathogen *Legionella pneumophila*

**DOI:** 10.1186/1471-2180-10-54

**Published:** 2010-02-19

**Authors:** Xiang-hui Li, Yong-lun Zeng, Ye Gao, Xiao-cong Zheng, Qin-fen Zhang, Shi-ning Zhou, Yong-jun Lu

**Affiliations:** 1 Department of Biochemistry, School of Life Sciences, Sun Yat-sen University, Guangzhou 510275, China

## Abstract

**Background:**

*Legionella pneumophila*, the intracellular bacterial pathogen that causes Legionnaires' disease, exhibit characteristic transmission traits such as elevated stress tolerance, shortened length and virulence during the transition from the replication phase to the transmission phase. ClpP, the catalytic core of the Clp proteolytic complex, is widely involved in many cellular processes via the regulation of intracellular protein quality.

**Results:**

In this study, we showed that ClpP was required for optimal growth of *L. pneumophila *at high temperatures and under several other stress conditions. We also observed that cells devoid of *clpP *exhibited cell elongation, incomplete cell division and compromised colony formation. Furthermore, we found that the *clpP*-deleted mutant was more resistant to sodium stress and failed to proliferate in the amoebae host *Acanthamoeba castellanii*.

**Conclusions:**

The data present in this study illustrate that the ClpP protease homologue plays an important role in the expression of transmission traits and cell division of *L. pneumophila*, and further suggest a putative role of ClpP in virulence regulation.

## Background

*Legionella pneumophila*, a Gram-negative, intracellular bacterial pathogen, is the opportunistic agent responsible for a severe form of pneumonia named Legionnaires' disease and the less severe flu-like Pontiac fever [1,2]. The remarkable capability of *L. pneumophila *to colonize a wide range of natural protozoa and mammalian host cells is mostly attributed to its unique Type IVB secretory system (T4BSS) whose components are encoded by the *dot *(defect in organelle trafficking) and *icm *(intracellular multiplication) genes [3-6]. *L. pneumophila *uses the Dot/Icm apparatus to inject effectors into the host cells to promote invasion and to modulate organelle trafficking, which in turn leads to formation of replication-permissive endosomes [7-9].

Similar to a variety of microbes, *L. pneumophila *undergoes a life cycle characterized by a biphasic conversion between a vegetative replicative form and a non-replicating, infectious and stress resistant transmissive form. On one hand, bacteria cultured in broth to either exponential or stationary phase display many similar attributes shared by the replicative and transmissive forms, respectively [10,11]. For example, upon the transition from exponential phase to stationary phase, *L. pneumophila *becomes more infectious and more resistant to various stresses [12]. Furthermore, *L. pneumophila *in stationary phase also displays shortened cell body, flagellin expression, pigment accumulation and reduced sodium sensitivity. These attributes, together with virulence markers such as cytotoxicity, intracellular growth and phagocytosis, are recognized as the transmission traits of *L. pneumophila *[11,13]. On the other hand, the *in vitro*-cultured stationary-phase *L. pneumophila *can achieve further differentiation to the cyst-like, hyper-infectious and resilient mature intracellular form (MIF) in aquatic environment or in specific mammalian cell lines. MIF is considered as an "*in vivo *stationary-phase form" while owning different outer membrane structure and protein composition compared with the stationary-phase form [14,15]. In addition, an *in vivo *transcriptome of *L. pneumophila *was performed and exhibited the genes strongly induced in intracellular replicative or transmissive phase, respectively, which also revealed several virulence or transmission related genes specially induced intracellularly, confirming the dissimilarity between the *in vitro*- and *in vivo*- transmissive/stationary phase [16].

A complicated gene network has been implicated in the regulation of transmission traits in *L. pneumophila*. For example, the sigma factor RpoS, the two-component system LetA/LetS, and the quorum sensing regulator LqsR have all been shown to facilitate the expression of transmission traits [10,11,13,17,18]. CsrA, a global repressor of transmission [19], also appears to be tightly regulated by several factors such as PmrA (positive regulator of several Dot/Icm-translocated effector proteins) and *rsm*YZ (two non-coding RNAs) [20,21]. In addition, CpxR has been found to activate transcription of several genes encoding components of the Dot/Icm complex as well as several Dot/Icm-translocated effectors [22,23]. The concerted action of these regulators not only contributes to the display of transmission traits, but also plays a vital role in the re-entry into the replicative phase [11,13,19,20,24].

Proteolysis of detrimental and misfolded proteins is critically important for protein quality control and cellular homeostasis [25-27]. Four classes of energy-dependent protease systems have been identified throughout prokaryotes: ClpAP/XP, ClpYQ (also named HslUV), FtsH and Lon. ClpP and ClpQ, the catalytic cores of the proteases, require Clp ATPase chaperones for the recognition and unfolding of substrates; on the other hand, in FtsH and Lon, a single polypeptide contains both ATPase and proteolytic activity [26,28]. The ClpP protease and Clp ATPase, which are widely distributed and highly conserved in various bacteria species as well as mitochondria and chloroplasts of eukaryotic cells [27,29,30], have been demonstrated to function in the regulation of stress response, sporulation and cell division [31,32]. For example, ClpXP is responsible for the degradation of RpoS, the sigma regulator of stress response in *E. coli *[26]. In *Salmonella enterica *serovar typhimurium, loss of ClpXP has been shown to result in the over-expression of *fliA *and *fliC*, which in turn induced a hyperflagellate phenotype [33]. In *Bacillus subtilis*, ComK/S, the two-component regulator of competence and sporulation, are tightly controlled by the successive binding and degradation mediated by MecA and ClpCP [26]. ClpP also seems to regulate virulence in many pathogens such as *Listeria monocytogenes, Streptococcus pneumoniae *and *Staphylococcus aureus *[31,34-36]. Finally, ClpP has been demonstrated to play a role in the biofilm formation [36-38].

As a ubiquitous bacterium in aquatic environment, *L. pneumophila *encounters numerous stresses such as elevated temperature, low pH and starvation during both planktonic existence and intracellular replication [11,12]. We hypothesized that a rapid response to a changing environment might require an uncharacterized proteolytic system in *L. pneumophila*. In the present study, we explored the role of *L. pneumophila *ClpP in growth, stress tolerance, cell morphology and virulence to amoebae host. We demonstrate that ClpP affects several *L. pneumophila *transmission traits and cell division, and ClpP might play an important role in virulence regulation.

## Results

### *clpP *homologue is required for optimal growth of *L. pneumophila *at high temperatures

**Figure 1:**
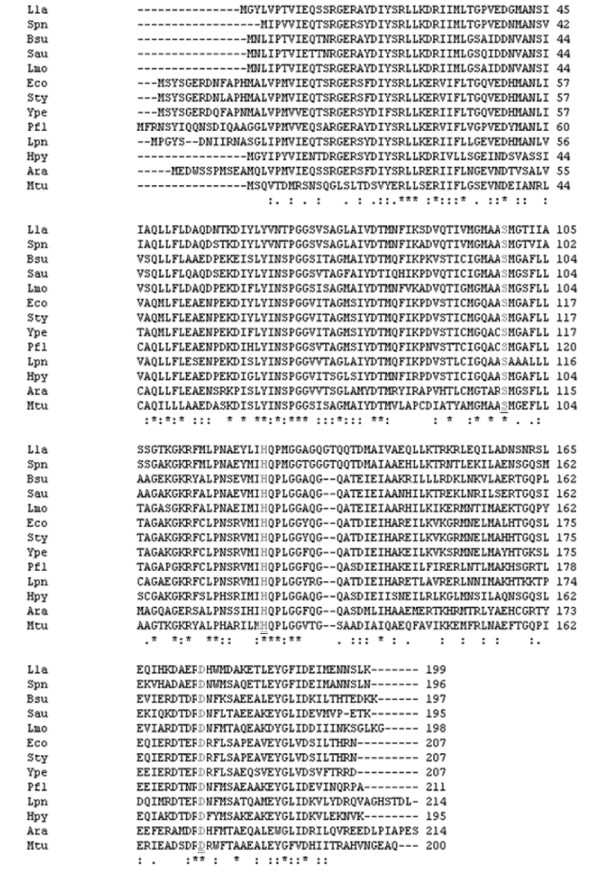
**Sequence alignment of the putative ClpP from *L. pneumophila *with other prokaryotic ClpP proteins**. Numbers indicate the positions of amino acids in the sequences, and dashes show gaps inserted for an optimal alignment. Identical or similar residues are labeled with asterisks or periods, respectively. The highly conserved catalytic Ser-110, His-135 and Asp-184 are shown as light color. Lla, *Lactococcus lactis*. Spn*, Streptococcus pneumoniae*. Bsu, *Bacillus subtilis*. Sau, *Staphylococcus aureus*. Lmo, *Listeria monocytogenes*. Eco, *Escherichia coli*. Sty, *Salmonella enterica *serovar typhimurium. Ype, *Yersinia pestis*. Pfl, *Pseudomonas fluorescens*. Lpn, *Legionella pneumophila*. Hpy, *Helicobacter pylori*. Ara, *Agrobacterium radiobacter*. Mtu, *Mycobacterium tuberculosis*.

**Figure 2:**
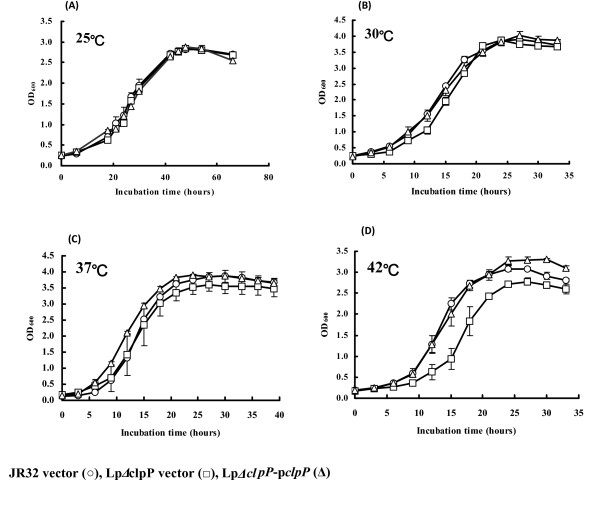
**The growth curves of *L. pneumophila *wild-type JR32, the Lp*ΔclpP *mutant, both harboring the vector pBC(gfp)Pmip, and the complemented strain Lp*ΔclpP*-p*clpP***. Overnight cultures of mid-exponential bacterial cells were diluted into fresh medium and then incubated at (A) 25°C, (B) 30°C, (C) 37°C, and (D) 42°C, respectively. Growth was monitored by OD_600 _at various time points. Points indicate mean values and error bars indicate standard deviations of three experiments.

In *L. pneumophila*, the *lpg1861 *sequence was predicted to encode a putative ClpP homologue. The product of *lpg1861 *consists of 215 amino acids and contains a highly conserved three-residue sequence Ser-His-Asp (Figure 1) that was previously reported as the proteolytic triad site of *E. coli *ClpP [27,39,40]. To investigate the physiological role of *clpP *homologue in *L. pneumophila*, we constructed a *clpP*-deficient mutant by non-polar deletion of a 519 bp internal fragment encompassing the coding sequence for Ser-His-Asp. We first determined the impact of *clpP *on growth. As shown in Figure 2, the growth curves of WT, the Lp*ΔclpP *mutant, and the constitutive complemented strain Lp*ΔclpP*-p*clpP*, were similar at 25°C, 30°C and 37°C (Figure 2A to 2C), demonstrating that *clpP *is not required for optimal growth at lower temperatures. However, the Lp*ΔclpP *mutant strain exhibited impaired growth at 42°C relative to the other two strains (Figure 2D), indicating an important role of *clpP *homologue for optimal growth of *L. pneumophila *at high temperatures.

### *clpP *homologue is required for stress tolerance in stationary phase

**Figure 3:**
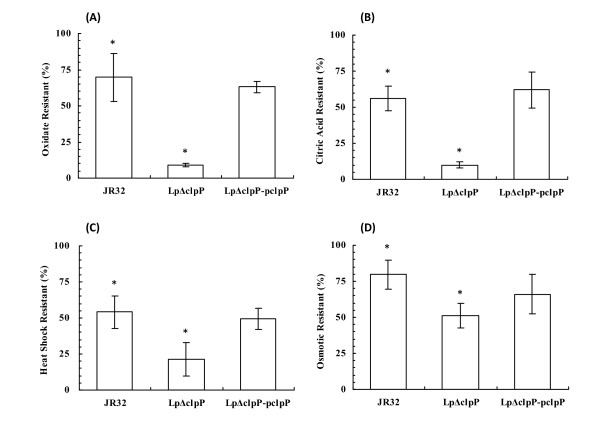
**Impaired stress tolerance of the *L. pneumophila *Lp*ΔclpP *mutant during stationary phase**. Overnight cultures of different strains were inoculated into fresh medium and grew to stationary phase (OD_600 _from 3.5 to 4.5), and the cells were then treated with (A) 1 mM H_2_O_2 _for 30 minutes. * *p *< 0.05, (B) pH 4.0 citric acid for 30 minutes. * *p *< 0.01, (C) 57°C heat shock for 20 minutes. * *p* < 0.05, or (D) 0.3 M KCl for 1 hour. * *p* < 0.05. The experiments were carried out in triplicate.

*L. pneumophila *can respond to various environmental stresses and cope with harsh conditions while entering eukaryotic hosts [12,41]. To assess whether *clpP *homologue may be involved in stress response, the above three strains were grown to logarithmic or stationary phase and exposed to various stress conditions. When the logarithmic-phase cells were exposed respectively to low pH, hydrogen peroxide, potassium chloride, and heat shock, the survival rates of all three strains were similar and lower than those of the stationary-phase cells (data not shown). When treated with pH 4.0 citric acid for 30 minutes, WT JR32 cells in stationary phase exhibited approximately 70% survival rate. However, only about 10% of Lp*ΔclpP *mutant cells survived (Figure 3A). Such a deficiency was rescued in the Lp*ΔclpP*-p*clpP *strain (Figure 3A). This result indicated that the deletion of *clpP *impairs the ability of *L. pneumophila *to respond to low-pH conditions. Similar results were also obtained in oxidative stress assay (Figure 3B). When the cells were treated with 1 mM hydrogen peroxide for 30 minutes, the survival rate of the Lp*ΔclpP *mutant was 10 ± 2.0%, much lower than that of WT cells (56 ± 8.6%; Figure 3B). In contrast, Lp*ΔclpP*-p*clpP *cells displayed a CFU closely resembling that of WT cells (Figure 3B). Likewise, when cells were incubated in 57°C water bath for 20 minutes or treated with 0.3 M potassium chloride for 1 hour, the survival rate of Lp*ΔclpP *mutant was lower than that of WT and the complementation strain (Figure 3C and 3D), indicating that *clpP *is also required for responses to heat shock and osmotic stress. Collectively, these results indicate that ClpP homologue is involved in tolerance to multiple stresses in stationary-phase *L. pneumophila*.

### *clpP *homologue is required for normal cell division of *L. pneumophila*

**Figure 4:**
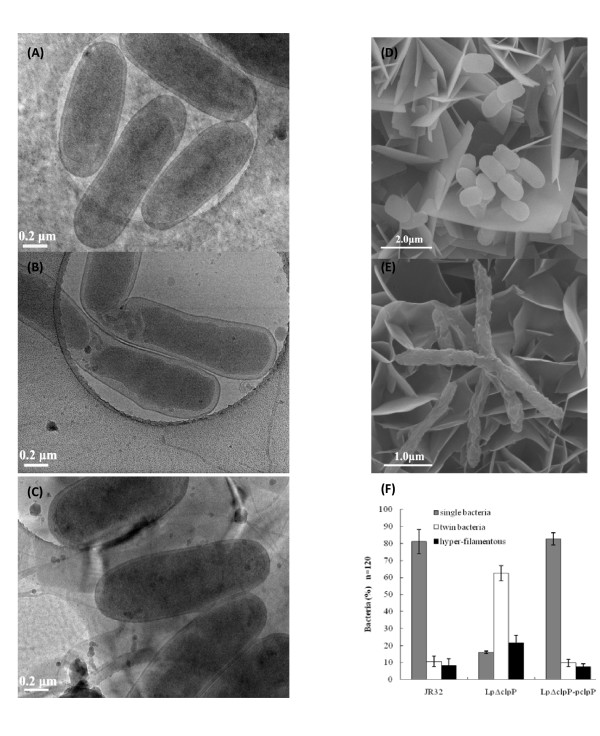
**Electron microscopy of stationary-phase *L. pneumophila *cells revealed cell elongation and abnormal division in the Lp*ΔclpP *mutant**. Cyro-TEM of (A) JR32, (B) Lp*ΔclpP *and (C) Lp*ΔclpP*-p*clpP *and SEM of (D) JR32 and (E) Lp*ΔclpP *were carried out. Bar for (A), (B) and (C), 0.2 μm; Bar for (D), 2.0 μm; Bar for (E), 1.0 μm. (F) The percentages of normal and abnormal cells under cyro-TEM in the three *L. pneumophila *strains. Shown are the averages and standard deviations of three independent counts and the number of cells for each count is about 120 (n = 120).

During stress tolerance assays, Lp*ΔclpP *generally exhibited 1.5- to 3-fold lower colony formation efficiency compared with WT JR32 on BCYE plates (data not shown). However, all three *L. pneumophila *strains appeared to have similar growth rates at 37°C, 30°C and 25°C (Figure 2A to 2C), thus excluding significant reduction in the number of living Lp*ΔclpP *cells. Previously, ablation of Clp protease activity has been shown to lead to abnormal cell wall formation or incomplete cell division in several Gram-positive bacteria [32]. To examine the morphology of Lp*ΔclpP *mutant cells under normal conditions, we performed cryo-transmission electron microscopy (cyro-TEM). Cells in stationary phase were frozen-hydrated by liquid nitrogen and directly observed at -172°C, and we found that Lp*ΔclpP *cell surface was surprisingly indistinguishable from that of the WT cells (Figure 4A and 4B), contrary to our results obtained by scanning electronic microscopy (SEM) (Figure 4D and 4E), indicating that ClpP deficiency did not affect cell wall architecture under normal growth conditions.

The combined results of SEM and cyro-TEM showed that unlike the "plump cocoid" shape of the WT or complemented strains, stationary-phase cells deficient in *clpP *were elongated and incapable to divide normally (Figure 4A to 4E). Furthermore, around 62% of Lp*ΔclpP *cells were twins, 23% were hyper-filamentous, and only 15% of cells were single (Figure 4F). In contrast, around 8% of WT JR32 cells were hyper-filamentous, and approximately 11% of cells were "twins" (Figure 4F). The abnormal cell morphology was also reversed by complementation (Figure 4C and 4F). These results together suggest that deletion of *clpP *lead to abnormal cell division and consequently aberrant cell morphology in *L. pneumophila*.

### The Lp*ΔclpP *mutant is sodium tolerant

**Figure 5:**
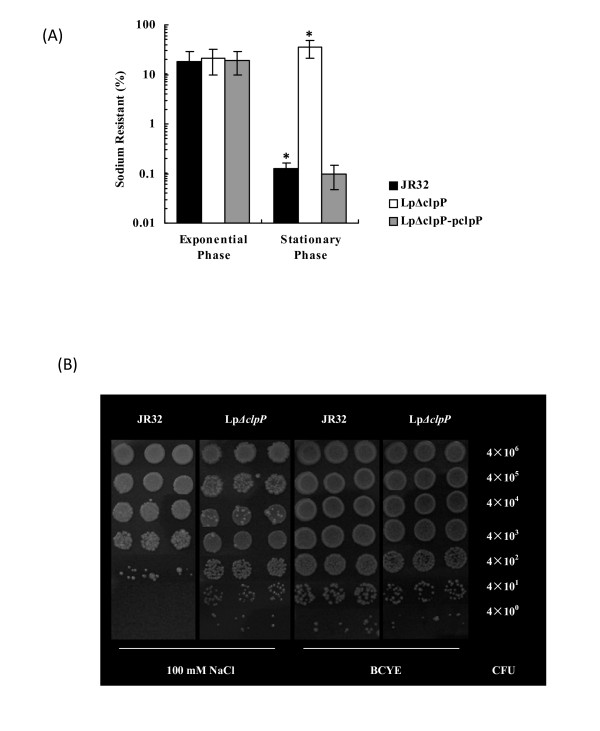
**Sodium tolerance of *L. pneumophila *Lp*ΔclpP *mutant was enhanced**. (A). Overnight bacterial cultures in mid-exponential phase were inoculated into fresh medium and grew to exponential phase (OD_600 _from 1.0 to 1.5) or stationary phase (OD_600 _from 3.5 to 4.5), then the CFU was determined by plating duplicate samples of JR32 (black bars), Lp*ΔclpP *mutant (white bars), and complemented strain (gray bars) on BCYE and BCYE containing 100 mM NaCl. The experiment was carried out in triplicate. * *p *< 0.01. (B). For direct visualization, different dilutions of stationary-phase JR32 and Lp*ΔclpP *cells were also spotted onto plates in triplicate.

Stationary-phase *L. pneumophila *cells have been shown to exhibit sodium sensitivity [42,43]. It has been proposed that the assembly of virulence factor translocation apparatus, such as the Dot/Icm T4SS complex, allows high levels of sodium to diffuse into the cytoplasm, which is lethal to the cells [44]. To investigate whether ClpP homologue also affected sodium sensitivity of *L. pneumophila*, JR32, Lp*ΔclpP *and Lp*ΔclpP*-p*clpP *strains were grown to exponential or stationary phase, diluted and plated in duplicate on BCYE or BCYE containing 100 mM sodium chloride, respectively. Different dilutions of stationary-phase JR32 and Lp*ΔclpP *cells were also spotted on the plates. In the presence of sodium, exponential-phase cells exhibited indistinguishable sodium sensitivity, irrespective of the genotype (Figure 5A). However, the Lp*ΔclpP *mutant displayed an approximately 300-fold higher resistance than JR32 in stationary phase (Figure 5A). The loss of sodium sensitivity as a result of *clpP *deletion was again reversed in Lp*ΔclpP*-p*clpP *(Figure 5A). The relationship between sodium resistance and *clpP *deletion was further confirmed by the plate-spotting assay (Figure 5B). Notably, while more resitant to sodium in both assays, Lp*ΔclpP *required two more days to form colonies on NaCl plates compared to JR32 (Figure 5; data not shown). Taken together, these results demonstrate that the deletion of *clpP *enhances the sodium resistance of *L. pneumophila *in stationary phase with a slower growth rate, implying a possible role of ClpP in virulence.

### Loss of *clpP *impaires *L. pneumophila *growth and its cytotoxicity against *A. castellanii*

**Figure 6:**
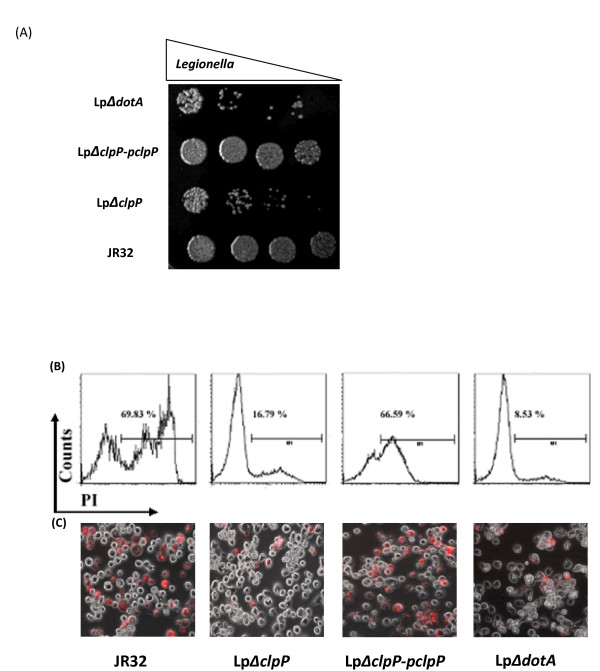
**The *L. pneumophila clpP *mutant was impaired in both cytotoxicity against amoebae *A. castellanii *and growth on amoebae plates**. (A) Growth of *L. pneumophila *Lp*ΔclpP *mutant in the amoebae plate test was impaired. *L. pneumophila *wild-type strain JR32, Lp*ΔclpP *mutant, *clpP *complemented strain or *dotA *mutant were spotted respectively in tenfold serial dilutions onto BCYE agar plates containing *A. castellanii*. The plates were incubated at 37°C for 5 days. (B) Cytotoxicity of L. pneumophila against amoebae A. castellanii was quantified by flow cytometry and (C) detected by PI staining 24 h post infection. The infection was performed using the wild-type strain JR32, Lp*ΔclpP *mutant, clpP complemented strain or dotA mutant at an MOI of 100. For fluorescence microscopy, amoebae cells in each well of 24-well plate were stained. The data shown are representative of at least two independent experiments.

To determine whether ClpP homologue may function in the virulence of *L. pneumophila*, we performed the amoebae plate test (APT) previously used to determine virulence [45]. The amoebae (*A. castellanii*) host cells were spread onto BCYE plates before stationary-phase *L. pneumophila *cells were spotted in 10-fold serial dilutions, and the plates were subsequently incubated at 37°C for 5 days. As shown in Figure 6A, WT JR32 and the complemented strain Lp*ΔclpP*-p*clpP *exhibited robust growth even at 10^-8 ^dilution when co-incubated with amoebae. However, Lp*ΔclpP *showed a growth defect resembling the phenotype observed in the negative control *ΔdotA *strain which was rendered completely avirulent by an in-frame deletion in the *dotA *gene [46]. As an additional control, cells were spotted onto the plates in the absence of amoebae, and no difference in growth was observed among the four strains (data not shown).

Cytotoxicity is an important virulent trait of *L. pneumophila *and correlates strongly with the function of the Dot/Icm T4SS [13,44,45,47]. We next tested whether *clpP *homologue may affect the cytotoxicity of *L. pneumophila *against *A. castellanii*. *L. pneumophila *strains were used to infect *A. castellanii *with an MOI of 100. 24 h post infection, cytotoxicity was assayed by PI staining and quantified by flow cytometry analysis [13,45]. As shown in Figure 6B, JR32 exhibited robust cytotoxicity (70% *A. castellanii *lethality), whereas Lp*ΔclpP *resulted in only 17% cell death, barely higher than that of the avirulent mutant *ΔdotA *(9% cell death). As expected, cytotoxicity was restored in the complemented strain Lp*ΔclpP*-p*clpP *(67% PI positive). These results were also confirmed by fluorescence microscopy (Figure 6C). Thus, it appeared that loss of *clpP *seriously impaires cytotoxicity against the amoebae host.

### Loss of *clpP *abolishes intracellular multiplication of *L. pneumophila *in *A. castellanii*

**Figure 7:**
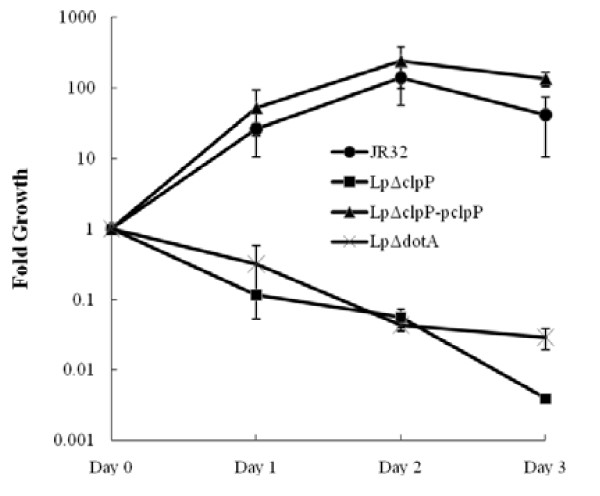
**Intracellular growth of *L. pneumophila *Lp*ΔclpP *mutant in *A. castellanii *was abolished**. *A. castellanii *cells were seeded onto 24-well plates and infected with *L.pneumophila *at an MOI of 10. At each time point indicated, amoebae cells were lysed and the CFU was determined by plating dilutions onto BCYE plates. The intracellular growth kinetics of JR32, Lp*ΔclpP *mutant, *clpP *complemented strain, and *dotA *mutant are shown. The infection assay was carried out in triplicate.

The above APT and cytotoxicity assays demonstrated an important role of *clpP *in virulence. Next, we examined whether *clpP *homologue also affected the intracellular replication of *L. pneumophila *in *A. castellanii*. Amoebae cells were infected with stationary-phase *L. pneumophila *at an MOI of 10. Under such conditions, the infection persisted for 3 days and multiplication was evaluated by plating the amoebae lysate onto CYE plates to quantify replication. As shown in Figure 7, JR32 and the complemented strain exhibited essentially identical replicative capability within *A. castellanii *cells. In contrast, both Lp*ΔclpP *and *ΔdotA *mutants showed significantly impaired multiplication. As a control, the Lp*ΔclpP *strain displayed normal growth at 30°C or 37°C in broth (Figures 2b and 2c).

Taken together, the adverse effects of *clpP *deletion on sodium tolerance, growth on APT, cytotoxicity and intracellular multiplication suggest that ClpP homologue might play an important role in virulence regulation of *L. pneumophila*.

## Discussion

In the current study, Lp*ΔclpP *was shown to exhibit reduced growth rate at high temperatures (Figure 2D) and impaired resistance to heat shock (Figure 3C) compared to the wild type. The Lp*ΔclpP *mutant also displayed impaired resistance to oxidative and low-pH conditions in stationary phase. As oxidative and acid stress are generally considered as harsh and detrimental to DNA [48,49], ClpP homologue may play an important role in *L. pneumophila *DNA repair, consistent with its demonstrated function in *E. coli *[50], *S. aureus *[51] and *Lactococcus lactis *[52]. However, while several previous studies have demonstrated growth defect as a result of ClpP deficiency over a broad temperature range [34,35,51], deletion of *clpP *appeared to compromise the growth of *L. pneumophila *only at higher temperatures (Figure 2A to 2C), suggestive of a more restricted role independent of cold response.

Attenuation of ClpP or Clp ATPase activities has been shown to lead to abnormal bacterial morphology such as filamentation, aberrant cell wall structure and irregular cell division [29,32,53-55]. Likewise, results from SEM and cyro-TEM revealed that the Lp*ΔclpP *mutant cells were elongated and defective in cell division (Figure 4). Furthermore, SEM results also implicated a role of *clpP *in stress tolerance in *L. pneumophila*. In contrast to the defective cell surface observed in SEM (Figure 4D and 4E), largely normal cell surface were found by cyro-TEM in Lp*ΔclpP *mutant cells grown under normal conditions (Figure 4A to 4C), suggesting that the chemical treatment during SEM sample preparation, not *clpP *deletion, may have resulted in the abnormal cell surface.

How ClpP affects cell division is not fully understood. In *C. crescentus*, degradation of the cell cycle repressor CtrA by the ClpXP complex has been shown to contribute to G_1_-S transition, and deletion of *clpP *blocked cell division [54]. In *B. subtilis*, cells overproducing MurAA, an enzyme in peptidoglycan biosynthesis and a substrate of the Clp protease, displayed a filamentous, undivided morphology reminiscent of the *clpP *mutant cells, suggesting that degradation of MurAA by ClpP might contribute to normal cell segregation [56]. Furthermore, through a ClpP-independent pathway, the *B. subtilis *ClpX appeared to modulate the assembly of the tubulin-like protein FtsZ [57], which is known to be a key process in the replication and division of Gram-negative bacteria [58]. Identification of the substrate(s) for ClpP may shed light on the regulatory mechanism of cell division in *L. pneumophila*.

ClpP proteolytic complexes play pivotal roles in protein degradation or modification [26,31,32]. During the transition of *B. subtilis *cells to stationary phase, ClpP degrades massive amounts of proteins previously produced in exponential growth phase [32]. Notably, *L. pneumophila *also undergoes a biphasic life cycle with mutually exclusive gene expression for replication or transmission [10,11]. While transiting from replication (exponential phase *in vitro*) to transmission (stationary phase *in vitro*), *L. pneumophila *activates an intricate network of regulators such as LetA/S, RpoS, PmrA, CpxR, *rsm*YZ, CsrA and LqsR [11,13,20,21,59]. As shown in our results, unlike the stationary-phase wild type which exhibits transmission traits, Lp*ΔclpP *mutant cells in stationary phase exhibit replicative forms such as reduced stress tolerance (Figure 2 and 3), cell elongation (Figure 4), enhanced sodium resistance (Figure 5), impaired cytotoxicity and growth on amoebae plates (Figure 6) and severely compromised intracellular multiplication in amoebae host (Figure 7). Thus, ClpP may play an important role in the transition from replication to transmission in *L. pneumophila*. On the other hand, several transmission traits are not affected by *clpP*-deletion such as pigment accumulation and transcription from the *flaA *(legionella flagellin coding) gene (our unpublished data), suggesting that the impact of ClpP on the transition to transmissive form in *L. pneumophila *is somewhat limited. Considering that ClpP always executes the post-transcriptional feedback regulation, and moreover, degrades the same substrates by cooperating with other proteases [26,31], one explanation to such a limitation is that the degradation of ClpP substrates could be compensated by other proteases in *clpP*-deletion mutant, thus ClpP cannot govern the transition just as the global regulators such as RpoS, CsrA or LetA/S in *L. pneumophila*.

ClpP plays prominent roles in virulence of various Gram-positive pathogens such as *S. aureus, S. pneumoniae *and *L. monocytogenes *[34-36,60]. Furthermore, ClpP was reported to control the levels of key virulence factors of type III secretory systems (T3SS) in certain pathogens such as *S. *typhimurium and *Yersinia pestis *[61,62]. Recently, it was reported that loss of ClpP attenuated the virulence of *Helicobacter pylori*, a pathogen owning type IV secretory system (T4SS) [63]. It is interesting that *clpP*-deletion severely compromised the *L. pneumophila *infection against amoebae host (Figure 6 and 7). In our results, the sodium resistance exhibited by Lp*ΔclpP *mutant (Figure 5), which is a phenotype shared by the mutants without functional Dot/Icm T4SS [48,64], together with the comparable decline in intracellular multiplication observed in Lp*ΔclpP *and *ΔdotA *mutants (Figure 7), suggest a role of ClpP in T4SS-dependent virulence through degrading a repressor or activating an up-regulator of the substrate(s) of ClpP. One possibility is that the ClpP protease has a major impact on the expression or function of Dot/Icm T4SS in *L. pneumophila*. Another possibility is that ClpP might be required for the expression of some T4SS substrates. In this case, loss of ClpP would also severely attenuate the intracellular growth even if the T4SS is intact, just as the case of *L. pneumophila *Sigma S factor (RpoS) [59]. Thus, identification of the substrate(s) of ClpP, which is currently underway in our laboratory, would help to discern the underlying relationship between ClpP and T4SS-dependent virulence in *L. pneumophila*.

## Conclusions

In summary, our study shows that the *L. pneumophila *ClpP homologue is required for cell division and several transmission traits including stress tolerance, cell shortening, sodium sensitivity, cytotoxicity, growth on amoebae plates and intracellular multiplication. The study further suggests that the ClpP homologue might be important for virulence regulation of *L. pneumophila*.

## Methods

### Cells and reagents

The bacterial strains, plasmids and primers used in this work are listed in Table 1. *Legionella pneumophila *strains were cultured on buffered charcoal yeast extract (BCYE) plates, or in *N*-(2-acetamido)-2-aminoethanesulfonic acid (ACES)-buffered yeast extract (AYE) medium, supplemented with 5 μg chloramphenicol ml^-1 ^if necessary [65]. *Escherichia coli *strains were cultured in Luria-Bertani (LB) agar plates or broth, supplemented with 30 μg chloramphenicol ml^-1 ^or 100 μg ampicillin ml^-1^. *Acanthamoeba castellanii *(ATCC 30234) was grown in proteose yeast extract glucose medium (PYG) at 30°C [66]. Bacto yeast exact and proteose peptone were obtained from Becton Dickinson Biosciences. All other reagents were from Sigma, unless specified otherwise.

**Table 1:** Bacterial strains, plasmids and oligonucleotides used in this study.

Strain, plasmid or primer	Phenotype, genotype or sequence	Reference or source
***E*.*coli *strains**		
DH5α	F^- ^*endA1 hsdRI7 *(r_k _^- ^m_k _^+^) *supE44 thi-1*λ^- ^*recA1 gyrA96 *(Nal^r^) *relA1 Δ *(*lacZYA-argF*)*U169 deoR *φ 80d*lacZ Δ *M15	Lab collection
DH5α*λpir*	DH5α transduced with λ*pir*	[69]
***L. pneumophila *strains**		
JR32	Virulent *L. pneumophila *serogroup 1, strain Philadelphia, salt-sensitive isolate of AM511	[43]
Lp*ΔclpP*	JR32 with *clpP *deletion	This study
Lp*ΔclpP*-p*clpP*	Lp*ΔclpP *containing p*clpP*	This study
JR32-pBC	JR32 containing pBC(gfp)Pmip	This study
Lp*ΔclpP*-pBC	Lp*ΔclpP *containing pBC(gfp)Pmip	This study
Lp*ΔdotA*	JR32 with *dotA *deletion	Lab collection
		
**Plasmids**		
pRE112	Mobilizable suicide vector for construction of gene knockouts in G^- ^bacteria, *oriT oriV *sacB Cm	[69]
pMD18-T	cloning vector, Ap	TaKaRa
pBC(gfp)Pmip	ColE1 *ori *Cm P*mip gfpmut2*	[70]
pRE*ΔclpP*	pRE112::*clpP *for *clpP *deletion	This study
p*clpP*	pBC(gfp)Pmip containing *clpP *under the control of *mip *promoter	This study
		
**Primers**		
P_XC-F1_	AGAGAGCTCCTGCCAGTAGGTCCTATAAG	This study
P_XC-R1_	TATGACATACAAGTTGCTGGACATTCTAC	This study
P_XC-F2_	CAACTTGTATGTCATAGGAACGCTCACC	This study
P_XC-R2_	GATGGTACCTGGGAAAATTGACAAACCGT	This study
P_XH-clpPF_	TGGTGGAAGCTTTAGGAGTATCTAGCAAAGTTATAAGTC	This study
P_XH-clpPR_	TGGTGGTCTAGATGAGAAAAAAGGAGAGTAAGC	This study

*Abbreviations: Ap, ampicillin resistant; Cm, chloramphenicol resistant; sacB, sucrose sensitive.

### DNA manipulation and chromosomal in-frame deletion

DNA manipulations were performed according to standard protocols [67]. All restriction enzymes were purchased from New England Biolabs. Pfu or Taq DNA polymerases were from TaKaRa. Purification of plasmids and genomic DNA was performed according to the manufacturer's instructions (Qiagen).

The in-frame deletion of *clpP *was performed by a non-polar strategy as described [68]. Briefly, upstream and downstream flanking sequences of *clpP *were amplified by PCR using the P_XC-F1_/P_XC-R1 _and P_XC-F2_/P_XC-R2 _primer pairs, respectively. The PCR products were mixed and then used as templates for the subsequent fusion PCR using the P_XC-F1_/P_XC-R2 _primers. Fusion PCR products were digested with *Kpn*I and *Sac*I and sub-cloned into the pRE112 suicide vector [69], yielding plasmid pRE*ΔclpP*. Allelic exchange was performed as follows. Briefly, pRE*ΔclpP *was introduced into the wild-type (WT) JR32 strain by electroporation and chloramphenicol^R+ ^colonies were selected on BCYE-Cm plates. Transformants were inoculated into AYE and then incubated on BCYE containing 5% sucrose for 3 days at 37°C to select for strains devoid of the vector backbone. Positive colonies were confirmed by PCR and sequencing.

### Complementation assay

A ColE1-type plasmid pBC(gfp)Pmip, carrying an enhanced GFP gene (gfpmut2) whose transcription was controlled by Pmip, the promoter of the Legionella-specific *mip *(macrophage infectivity potentiator) gene, was used for the *clpP *compensation experiment [70,71]. As a control, the transcriptional activity of the *mip *promoter was not discernibly affected by the loss of *clpP *in JR32 (data not shown). pBC(gfp)Pmip was digested with *Xba*I and *Hin*dIII to remove the *gfp. *Sequences of *clpP *were amplified by PCR using the P_XH-clpPF _and P_XH-clpPR _primers, and the products were digested with *Xba*I and *Hin*dIII. The digestion products were ligated with the vector. The constructed plasmid p*clpP *was then electroporated into Lp*ΔclpP*, providing exogenous expression to compensate for the loss of *clpP*.

### Growth experiments

The growth experiments were conducted using three *L. pneumophila *strains, including JR32 and the *clpP *deficient Lp*ΔclpP *derivative, both harboring the pBC(gfp)Pmip vector, as well as the complemented strain Lp*ΔclpP*-p*clpP*. These strains were first grown in 5 ml AYE for about 20 h. The cultures were expanded into 30 ml AYE in flasks, incubated to mid-exponential phase [optical density at 600 nm (OD_600_) 1.5-2.5], then diluted into new flasks to similar optical densities at approximate OD_600 _0.2. These new cultures were then incubated at 25°C, 30°C, 37°C, and 42°C, respectively. OD_600 _was determined by Beckman Du-530 at various time points.

### Stress resistance assays

Resistance to stresses was measured as previously described [12,65], with minor modifications. Cells from 1 ml broth cultures were centrifuged at 5,000 g for 5 min, and resuspended in AYE supplemented with 1 mM hydrogen peroxide, 0.1 M citric acid at pH 4.0, or 0.3 M potassium chloride, respectively. 30 min later (1 h for osmotolerance assay), cells were washed by centrifugation and resuspended in AYE. Cultures were subsequently serially diluted in water, plated on BCYE for colony forming unit (CFU) counting.

In heat resistance assays, cells from 1 ml broth cultures were centrifuged at 5, 000 g for 5 min and then resuspended in AYE. Samples for heat-shock were placed in a 57°C water bath for 20 min, with the control in a 37°C water bath. Cells were washed and serially diluted in AYE, and spread on BCYE for CFU counting.

Stress resistance was calculated as [(stressed sample CFU ml^-1^)/(control sample CFU ml^-1^)] × 100.

### Sodium sensitivity assay

Sodium sensitivity assay was performed as previously described [65]. Briefly, cells from 1 ml broth cultures were centrifuged at 5, 000 g for 5 min and then resuspended in AYE. Subsequently, the cell suspensions were serially diluted in water, and spotted on BCYE and BCYE containing 100 mM NaCl or spread on plates for CFU counts. Sodium sensitivity was calculated as [(BCYE-100 mM NaCl CFU ml^-1^)/(BCYE CFU ml^-1^)] × 100.

### Electron microscopy

For scanning electron microscopy (SEM), *L. pneumophila *cells in exponential or stationary phase were collected by centrifugation at 5,000 g for 2 minutes, and then washed 3 times with 1×PBS. After being fixed by 2% glutaraldehyde (pH 7.4) and 1% osmium tetroxide followed by dehydration in a graded ethanol series and isoamyl acetate embedding, the cells were dried by using a critical point drying method, and mounted on aluminum stubs and shadowed with gold. For visualization, a scanning electron microscope (Hitachi/Oxford S-520/INCA 300) was used at 10 kV.

For Cryo-transmisson electron microscopy, *L. pneumophila *cells were collected and washed using the same method as above. The cells were then resuspended in 1×PBS and 4 μl sample aliquots were directly applied to a holey carbon film grid (R3.5/1 Quantifoil Micro Tools GmbH, Jena, Germany), followed by blotting with filter paper (Whatman #1) for about 3 seconds. The grid was then immediately flash frozen by plunging into pre-cooled liquid ethane. The cryo-grid was held in a Gatan 626 Cryo-Holder (Gatan, USA) and transferred into TEM (JEOL JEM-2010 with 200 kv LaB_6 _filament) at -172°C. The sample was scanned and observed under minimal dose condition at -172°C. The micrographs were recorded by a Gatan 832 CCD camera at a nominal magnification of 10,000~ 50,000× and at the defocus of 3-5.46 μm.

### Amoebae plate test (APT)

APT was performed as previously described [45]. Briefly, *A. castellanii *cells were cultured in PYG medium for 3 days prior to the test. A medium change was carried out one day before the test. The amoebae cells were washed off from the tissue culture flask, collected by centrifugation at 2,000 rpm for 5 min and resuspended in PYG to a density of 2 × 10^6 ^ml^-1^. 2 × 10^6 ^*A. castellanii *cells were spread on BCYE agar plates, and incubated at room temperature overnight. Series of tenfold dilutions of stationary-phase bacterial cultures at a starting density of 1 OD_600 _(=10^9 ^cells ml^-1^) were prepared. 10 μl of each dilution were spotted onto the amoebae-CYET agar plates, and incubated at 37°C for 5 days.

### Cytotoxicity assay using *A. castellanii*

To determine cytotoxicity, 2.5 × 10^5 ^amoebae cells were infected by bacteria at a multiplicity of infection (MOI) of 100. 24 h post infection, propidium iodide (PI) was added to 3 mg ml^-1^. *A. castellanii *cells were detached from the wells and 2.5 × 10^4 ^infected amoebae per sample were analyzed using a FACSCalibur flow cytometer (Becton Dickinson) with a scatter gate adjusted for *A. castellanii *[13]. Excitation was at 458 nm and fluorescence was measured at 495 nm. The data were collected and analyzed using the CELLQUEST software (Becton Dickinson). For fluorescence microscopy, the infected amoebae cells in each well of 24-well plates were stained with PI, then observed in bright field or by epifluorescence with an inverse microscope (Zeiss Axiovert 200 M, 20 × objective).

### Intracellular growth in *A. castellanii*

For intracellular growth assays, exponentially growing *A. castellanii *were washed with Ac (*A. castellanii*) buffer, resuspended in HL5 medium, seeded onto a 24-well plate (2.5 × 10^5 ^per well) and were allowed to adhere for 1-2 h. *L. pneumophila *was grown for 21 h in AYE broth, diluted in HL5 and used to infect amoebae at an MOI of 10. The infection was synchronized by centrifugation at 440 g for 10 min, and the infected amoebae were incubated at 30°C. Thirty minutes post infection, extracellular bacteria were removed by washing 3 times with warm HL5 medium [13]. At the time points indicated, culture supernatant was removed and the amoebae cells were lysed with 0.04% Triton. The supernatant and the lysates were combined, and serial dilutions were prepared and aliquots were plated on CYE plates for CFU counting [72].

### Statistical analysis

Basic statistical analyses were performed using Excel, and one-way ANOVA was performed using SPSS followed by a post hoc Student-Newman-Keul's test. The alignment of amino acid sequences was performed using the online ClustalW2 http://www.ebi.ac.uk/Tools/clustalw2.

## Abbreviations

*dot*: defect in organelle trafficking; *icm*: intracellular multiplication; *mip*: macrophage infectivity potentiator; MIF: mature intracellular form; MOI: multiplicity of infection; APT: amoebae plate test

## Authors' contributions

XHL and YJL designed the experiments and drafted the manuscript. XHL performed the experiments. YLZ and YG participated in the design of the study and performed the amoebae infection analysis. XCZ carried out part of molecular cloning work. QFZ carried out the cyro- electron microscope observation. SNZ participated in designing the study and helped to draft the manuscript. All authors read and approved the final manuscript.
